# The use of haystacks by wolves may facilitate the transmission of sarcoptic mange

**DOI:** 10.1038/s41598-024-78026-w

**Published:** 2024-11-16

**Authors:** Katarzyna Bojarska, Blanka Orłowska, Wojciech Sobociński, Małgorzata Karczewska, Marta Kołodziej-Sobocińska

**Affiliations:** 1 Institute of Nature Conservation, Polish Academy of Sciences, A. Mickiewicza 33, Cracow, 31-120 Poland; 2 Department of Food Hygiene and Public Health Protection, Institute of Veterinary Medicine, Warsaw University of Life Sciences, Nowoursynowska 159, Warsaw, 02-776 Poland; 3 Faculty of Biology, University of Białystok, Ciołkowskiego 1J, Białystok, 15-245 Poland; 4 Białowieża National Park, Park Pałacowy 11, 17-230 Białowieża, Poland; 5 Mammal Research Institute, Polish Academy of Sciences, Stoczek 1, Białowieża, 17-230 Poland

**Keywords:** Anthropogenic resources, *Canis lupus*, Environmental transmission, Human-wildlife conflict, *Sarcoptes scabiei*, Ecology, Ecology, Environmental sciences, Risk factors, Diseases, Infectious diseases

## Abstract

Wildlife that use anthropogenic resources often come into conflict with humans, e.g. due to damaged property, habituation or transmission of pathogens, amongst them *Sarcoptes scabiei*, the aetiological agent of sarcoptic mange, an emerging panzootic skin disease. This study examines the use of haystacks intended for supplementary feeding of European bison (*Bison bonasus*) by wolves (*Canis lupus*) with sarcoptic mange and the potential role of this behaviour in skin parasite transmission and human-wolf conflict. Hay samples from the beds used by wolves were found to harbour *S. scabiei* mites, even several days after the last use. Our data demonstrate an unforeseen link between wild ungulate supplementary feeding and wolf behaviour that may lead to conflict, namely approaching human settlements. However, no negative interactions were observed between wolves and humans or domestic animals. The presence of *S. scabiei* mites in haystacks provides a potential for its human-facilitated environmental transmission among wildlife and to domestic animals.

## Introduction

The emergence of infectious diseases in wildlife poses a substantial threat to the conservation of global biodiversity^1^. In an era of global change, human activities such as hunting^2^, as well as habitat alterations^3–5^, supplementary feeding^6,7^, wildlife translocation^8^ and the introduction of pathogens carried by alien species^9,10^ or domestic animals^11^, have a strong influence on potential host population size, demography and behaviour. These factors play a significant role in the emergence of disease and its transmission in wildlife.

Human activity can accelerate disease spread throughout wildlife populations, and this can have cascading effects on ecological communities and ecosystems^12,13^. For example, long-term research revealed an outbreak of sarcoptic mange in a remote Andean protected area to have considerable effects on trophic cascades involving pumas (*Puma concolor*), vicuńas (*Vicugna vicugna*), condor (*Vultur gryphus*), and plant communities. A decrease in vicuńa density (herbivores) caused by the outbreak, led to a sharp decline in the occurrence of condors (scavengers) and a dramatic increase in plant cover in predation refuges, leading to transformation of the whole ecosystem^13^.

The need to minimize negative interactions between people and wildlife has been highlighted as a priority for conservation efforts^14^. Conflicts between humans and wild animals typically arise when wildlife is attracted to food or shelter associated with humans^15^, and may be aggravated by the risk of transmission of zoonotic diseases to people and domestic animals^16^. Transmission of parasites or infectious diseases from free-ranging animals to humans or pets has become a major area of human-wildlife conflict^17^. Infection with certain pathogens may decrease fear of humans in wildlife, thus encouraging them to approach settlements; for example, carnivores with rabies typically exhibit fearless behaviour^18^ and may attack humans^19^. In most cases, however, pathogens induce subtler behavioural modifications by worsening general body condition and nutritional status. For example, diseased and malnourished wildlife tend to feed on human-derived food, which may transform sites with anthropogenic resources into hotspots for pathogen transmission^7^.

One example of such a disease is sarcoptic mange: a global skin disease caused by the mite *Sarcoptes scabiei*, which has been reported in at least 148 species of mammals and is considered an emerging panzootic^20^. Epidemics of sarcoptic mange have been spreading rapidly throughout wildlife populations over broad geographical ranges and are associated with a sharp decrease in host density and substantial structural shifts in population dynamics^21,22^. The transmission of *S. scabiei* occurs by direct contact or through sharing an environment, i.e. through indirect or environmental transmission^20^. Indirect transmission is possible due to the mites being able to remain infectious away from the host, where the microclimate allows^23–25^. Natural burrows and dens have been reported as transmission sites for sarcoptic mange in various scenarios: in San Joaquin kit fox (*Vulpes macrotis mutica*)^25^, American black bear (*Ursus americanus*)^26^, in a carnivore community in Białowieża Primeval Forest^24^, and among bare-nosed wombats (*Vombatus ursinus*)^27^. However, a critical knowledge gap remains regarding the role that indirect transmission plays in the spread of sarcoptic mange^27^. As with other indirectly-transmitted pathogens, the interactions between host behaviour and the environmental reservoir represent a crucial link in disease transmission^28^. As such, it is essential to identify and monitor potential hotspots for the spread of this disease.

Sarcoptic mange mites burrow into the skin of their hosts causing acute or chronic infections accompanied by severe itching, hair loss and hyperkeratosis. Infection may also cause metabolic changes and impair thermoregulatory capacity, resulting in death by hypothermia and starvation^27,29^. Individuals with severe mange symptoms have been reported to change their behaviour, by altering their food habits or movement and activity patterns^30–32^.

Some of the behavioural effects caused by sarcoptic mange may lead to increased conflict with humans. Red foxes (*Vulpes vulpes*) and coyotes (*Canis latrans*) that carry sarcoptic mange select areas closer to human settlements and increase their use of anthropogenic resources^33–35^. Wolves (*Canis lupus*) are also often affected by sarcoptic mange^36^, but they are more likely to be involved in human-wildlife conflicts than smaller carnivores^14^. Wolves exhibit great behavioural flexibility that allows them to learn how to use anthropogenic resources and they become habituated to humans^37^, which may lead to wolf attacks^38^. In winter, wolves with mange suffer from considerable heat loss, display lower movement rates and shift to more diurnal activity^39^, which increases the chance of encounters with humans. However, no studies so far have found a link between sarcoptic mange and conflictive behaviour in wolves.

The present study examines the behaviour of wolves with mange seeking shelter in haystacks intended for supplementary feeding of European bison (*Bison bonasus*). It explores the presence of sarcoptic mange mites in the hay used by wolves and discusses the potential implications of such use of haystacks in the transmission of mange and human-wildlife conflict. We hypothesize that severe sarcoptic mange infections cause symptomatic wolves to seek shelter in haystacks. As a result, hay provided for supplementary feeding of ungulates may contain *S. scabiei* mites and become a potential source of sarcoptic mange infection.

## Materials and methods

### Study area

**Fig. 1:**
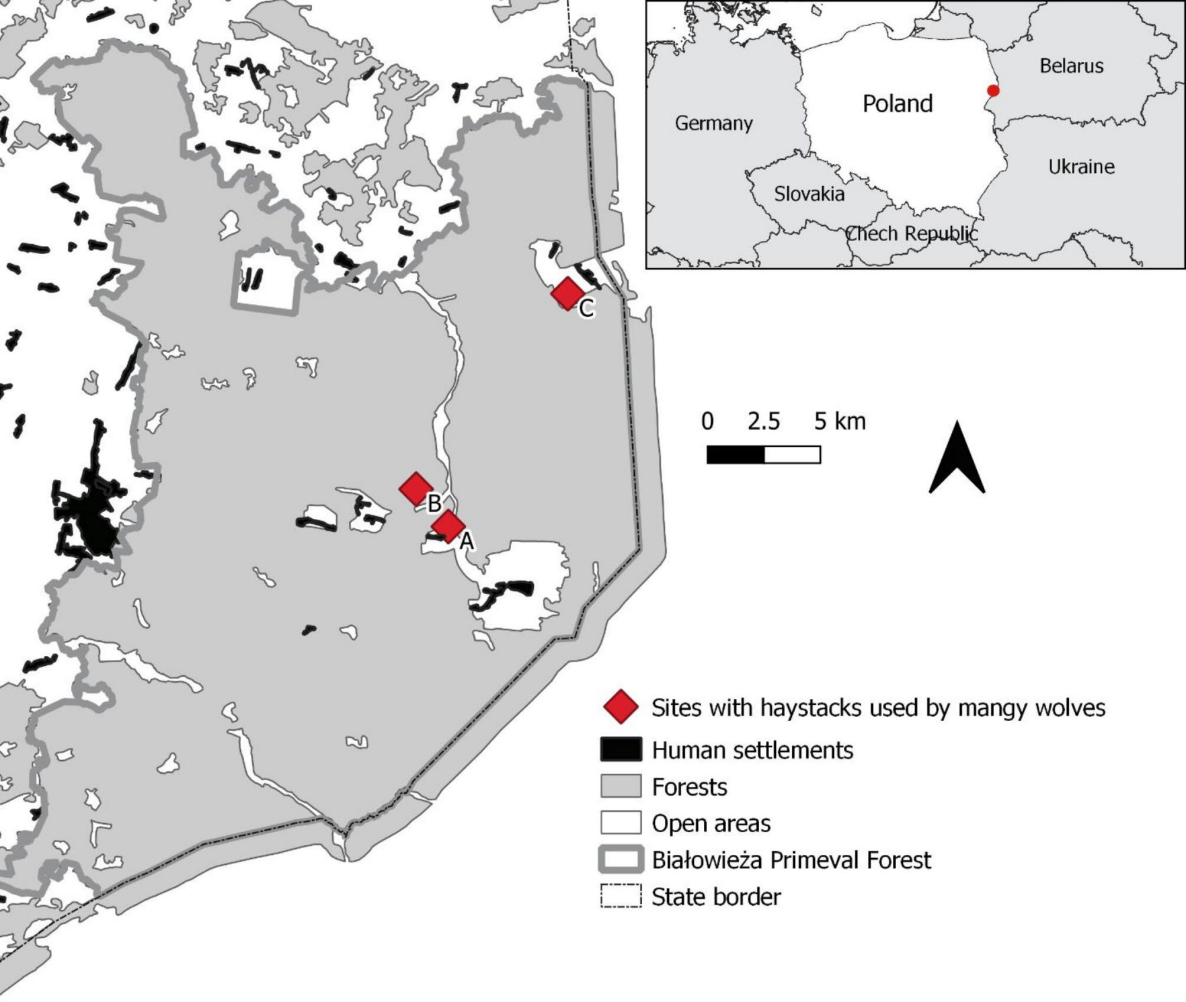
Location of the sites where wolves with mange used haystacks in the Białowieża Primeval Forest, Poland: in January 2022 (Pogorzelce, A), in March 2022 (Wolf Glade, B) and January 2024 (Stare Masiewo, C).

The study was carried out in the Białowieża Primeval Forest (BPF, north-eastern Poland, bordering with Belarus). The Polish part of the BPF (52° 29’–52° 57’ N, 23° 31’–24° 21’E) covers 650 km^2^ and is one of the best-preserved lowland deciduous forests in Europe. The forest consists of mixed deciduous woodlands (93.6% of the area), interspersed with open areas (glades with meadows and small villages, river valleys with sedges and reed marshes, Fig. 1). Human density is low: approx. 12 people per km^2^^40^. The study area is inhabited by well-preserved wild mammalian communities, with 13 species of carnivorous mammals and five species of ungulates^41^.

Most of the meadows in the area are located around the settlements, on the outskirts of BPF, and some smaller glades are scattered throughout the forest. The meadows and glades are mowed every year in response to open habitat conservation policies and for the management of European bison population, as stacked hay is often intentionally left for their feeding supplementation in winter.

Wolf behaviour was recorded in two periods: January to March 2022, and in January 2024. No wolves with acute mange-compatible symptoms were noted in winter 2022/23 (authors’ own data). Winter 2022 was a mild one, with mean monthly temperatures − 0.7, 1.6 and 0.8 ºC for January, February and March, respectively. Cumulative precipitations during those months were 56.3, 40.5 and 3.3 mm, respectively, and snow cover was recorded for a total of 16, seven, and three days in January, February and March. January 2024 was characterised by more severe weather, with a mean temperature of -3.2 ºC; 69.1 mm of precipitation and 30 days of snow cover (data obtained from the Institute of Meteorology and Water Management, https://danepubliczne.imgw.pl/data/). The climate change in BPF over the last decade follows similar patterns as the rest of central Europe, with increasing air temperatures and decreasing precipitation^42^.

During the study period, BPF was inhabited by at least six packs of wolves (authors’ unpublished data), and although continuous data on wolf numbers is not available, the number of packs seems to have increased over the last decades, as two to four packs were recorded in 1980–1990s^43^. Of the six packs detected, at least one was affected by mange, with juvenile individuals displaying skin clinical signs. In 2021/22, a pack counting at least 11 individuals included five to six individuals with visible mange symptomatology; in contrast, in 2023/24, a pack of at least eight individuals was seen to include four to five wolves with mange (authors’ unpublished data). Due to lack of telemetry or genetic data, it was not possible to confirm whether the packs studied in 2022 and 2024 consisted of the same breeding pair.

### Camera trapping and sample collection

Local inhabitants reported wolves using haystacks at three locations in BPF in January and March 2022 and in January 2024 (Fig. 1): in Pogorzelce village (52.7238 N, 23.8068 E, in January 2022, hereafter site A), in a forest glade (supplementary feeding site for European bison, hereafter site B, 52.7421 N, 23.7951 E, in March 2022) and in Stare Masiewo village (hereafter site C, 52.8218 N, 23.9171 E, in January 2024). There were two haystacks at each site. To evaluate the presence of infected wolves in the haystacks, a camera trap was placed at each site facing one or both haystacks. The cameras were set for continuous activity, and to record 30- or 60-second videos triggered by movement.

After the camera trapping was complete, we collected hay samples from the wolf beds in the haystacks to test for the presence of *S. scabiei* mites. We collected one hay sample (approx. 100 g) from the bottom side of each wolf bed, including the bottom surface and deeper layers (up to 10 cm deep). We additionally collected control samples from a randomly-chosen side of each used haystack, where no signs of animal resting were visible; these samples were intended to test whether the mites were only present in the wolf beds, or also in other parts of the haystacks. We used disposable nitrile gloves during sample collection, and changed them after the collection of each sample.

We collected deep skin scrapings and skin biopsies from three dead wolves with mange-compatible symptoms; all were found in the vicinity of sites B and C. We took the samples from the neck, sternum, groin, armpit, and the base of the tail. For each sample, around 0.5 cm^3^ of skin was taken, including all layers. In each case, the skin from suspected lesions was scraped with a sterile surgical blade.

#### Site A

**Fig. 2:**
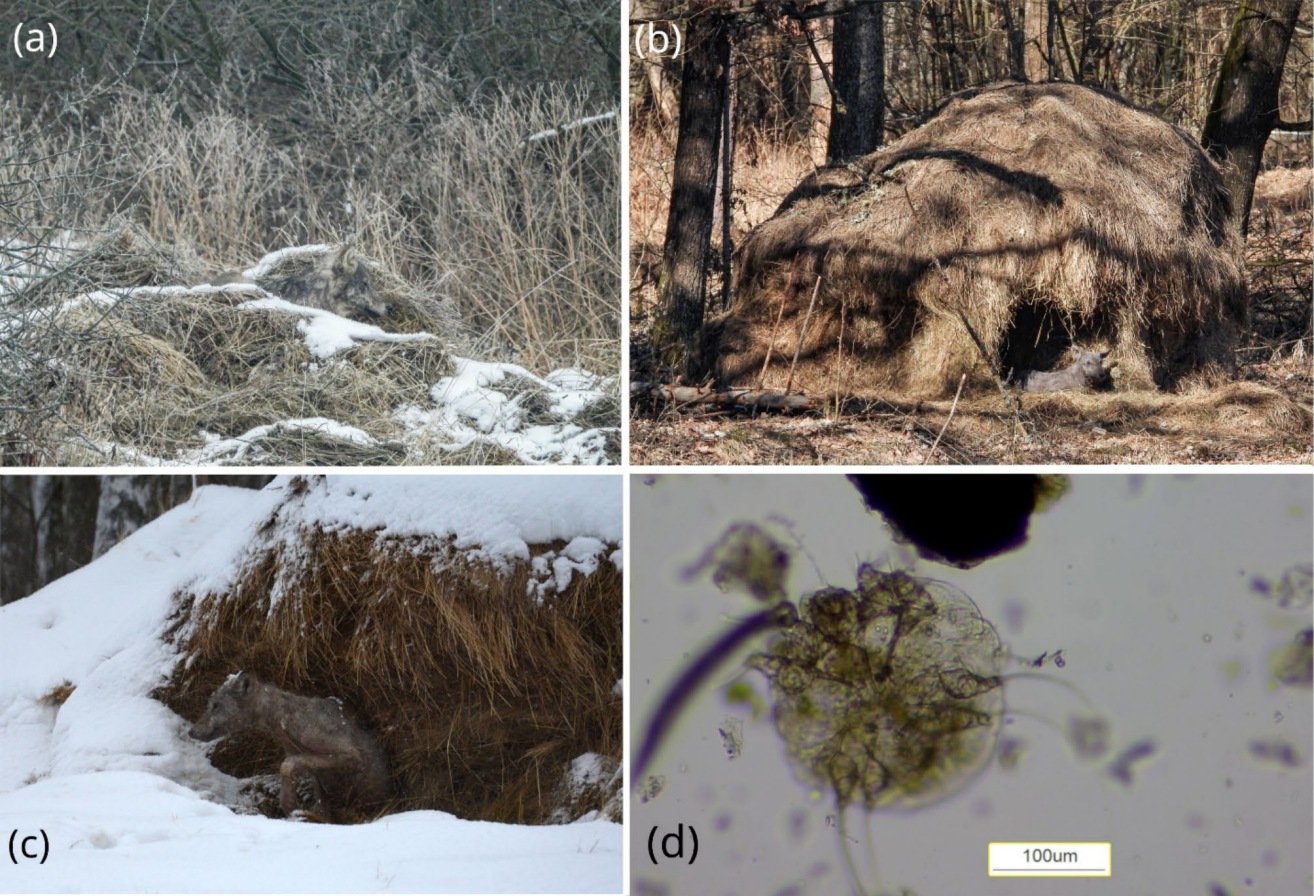
Wolves with visible symptoms of mange using haystacks at three sites in Białowieża Primeval Forest, Poland: (a) Site A in February 2022, (b) Site B in March 2022, and (c) Site C in January 2024; and (d) *Sarcoptes scabiei* mite obtained from a hay sample, 100x magnification (in sucrose solution). Pictures taken by: a, b – Adam Bochiński, c – Katarzyna Bojarska, d – Blanka Orłowska.

In January 2022, a pack of wolves, several of which exhibited clinical symptoms consistent with mange, was regularly observed by local inhabitants in the vicinity of settlements of site A (Fig. 2A., pers. comm. Adam Bochiński). The wolves were reported to seek shelter in two heaps of hay left after mowing the grass on meadows surrounding the village, 450 m from the closest houses. We placed a camera trap (Bolyguard SG520) facing both haystacks. The camera was active from 15 January to 28 February 2022 (44 camera trap-days). At the end of this period, we identified two clear wolf beds, one in each haystack. Two hay samples were then collected: one from each wolf bed.

#### Site B

In March 2022, wolves with clinical signs consistent with mange, were regularly observed using two well-formed haystacks left after mowing at site B (Fig. 2B), located approximately 2 km from site A. Due to the close proximity between sites A and B, it can be assumed that wolves observed at both sites belonged to the same pack. Although it was not possible to confirm the pack identity, wolves at site B appeared to manifest more severe symptoms (Fig. 2A and B).

We placed one camera trap (Denver WCS-5020) facing one of the haystacks for one night (March 22); it was not possible to monitor the haystacks for longer due to the high exposure of the camera and risk of theft. We placed an additional camera trap (Bolyguard SG520) on a wildlife trail leading to the glade with the haystacks, to allow longer-term (1–19 March 2022, i.e. 19 camera trap-days) monitoring of the wolves.

In April 2022, staff of the Białowieża National Park found a dead wolf approx. 300 m from the haystacks. A skin sample was taken from this individual to confirm the presence of *S. scabiei* mites. We collected eight hay samples at the beginning of May 2022, i.e. four from four beds (two beds in each haystack), and four control samples.

#### Site C

In January 2024, a pack of wolves with at least three individuals exhibiting mange symptoms, was regularly observed in and around settlements of site C. A local inhabitant reported some of wolves using two haystacks left for European bison (Fig. 2C). The haystacks were located 500 m from the closest buildings and 350 m from an enclosure where a herd of Hucul horses was pastured 24 h per day with no protection against wolves. We placed a Bolyguard SG520 camera trap facing both haystacks for one night; again, longer-term observation was not possible due to the risk of theft. Upon removal of the camera in the morning, we collected two hay samples from two wolf beds (one per each haystack) as well as two control samples. We took another set of hay samples two weeks later to confirm whether the mites were still present after this period of non-use; as one haystack had been destroyed by feeding, we only collected one sample from the remaining bed.

Two dead wolves were found while examining the haystacks at site C: one was found approx. 100 m from the haystacks on the day the camera trap was set, and another approximately 300 m from the haystacks two weeks later. Skin samples were collected from both individuals.

### Mite detection in hay samples

*S. scabiei* mites were detected by a combination of two modified conventional parasitological techniques: sedimentation and direct flotation^44,45^, designed especially for the purpose of this study. Each hay sample was soaked in 3 l of tap water for 15 min. The hay was then removed and the remaining water left for 24 h, after which the supernatant was decantated. Following this, direct flotation was performed with some modifications^46,47^; a 12 ml amount of the sediment from each sample was placed in a test tube and centrifuged at 1300 rpm for five minutes. Finally, sucrose solution (specific gravity 1.27) was added to make a bulging meniscus, which was covered with a coverslip for 10 min. Afterwards, the coverslip was placed on a glass slide and examined for the presence of mites under an OPTA-TECH LAB40 light microscope. These methods do not allow the viability or infectivity of mites recovered from the hay to be determined.

### Mite detection in skin samples from dead wolves

The contents of the skin scrapings were placed onto a microscope slide with three drops of 10% KOH, a coverslip was added and the samples were examined under 40× magnification for the presence of mites. To improve the chances of finding mites, the skin biopsies  (0.5 cm^3^ of skin ) were subjected to KOH skin digestion according to Tiffin et al.^48^, with modifications. Skin biopsies from the suspected lesions were divided into 1-mm thick pieces and incubated in 10% KOH at 37 °C for 24 h. The obtained digested material was centrifuged for five minutes at 1,300 rpm in 15 ml corning tubes. The supernatant was discharged, and the sediment was subjected to centrifugation-flotation in sucrose solution (specific gravity 1.27) and analysed under an optical microscope (10x, 40x magnification, as in conventional coproscopic methods)^46^. Mites were identified based on their morphology^49^.

## Results

### Site A

Camera trapping revealed that the two haystacks at site A were visited regularly by wolves. From 15 January to 16 February 2022, the presence of wolves was noted 21 times on nine days. 86% (*n* = 18) of the wolf recordings involved individuals with symptoms consistent with mange. Two of these 18 videos also included apparently healthy wolves together with the sick ones. The remaining 14% (*n* = 3) of videos involved either only apparently healthy wolves (one video) or the presence of symptoms could not be reliably assessed due to poor visibility (two videos). Based on the lesion patterns, at least two infected individuals could be identified; both were small and hence probably born in the previous year. The wolves demonstrated various behaviours, from resting in both haystacks and rolling in the hay to just crossing the field of view. Individuals without symptoms were recorded three times and were either passing by or standing and did not approach the haystacks.

52% (*n* = 11) of the wolf recordings were taken during the day, while the remaining 10 were taken at night. The videos confirmed the presence of red foxes three times, including one visually-healthy individual and two individuals with clinical signs of sarcoptic mange, but no foxes were observed to use the haystacks. *S. scabiei* mites were detected in one of the two wolf beds (Table 1).

### Site B

Based on the camera trap recordings, we confirmed that at least two individuals with severe mange symptoms used the beds in deep cavities in the walls of haystacks at site B. Between 1st March and 19th March 2022, infected wolves (probably two individuals) were recorded 12 times walking on the trail close to the haystacks; however, no apparently healthy individuals were recorded. 75% of the recordings on the trail were taken during the day.

On the night of 22nd March 2022, the camera facing the haystack recorded an individual with severe mange symptoms resting in a bed for approx. 12 h (from 6 pm to 6 am), with two short breaks of five and eight minutes. During that night, the site was visited also by other wolves: one recording showed an individual passing by the haystack (the presence of symptoms could not be discarded due to poor visibility), and two other recordings included only close clear voices of howling and growling wolves: no specific wolf individuals were recorded. No *S. scabiei* mites were found in any of those wolf beds (Table 1); however, *S. scabiei* mites were identified in the skin biopsies and scrapings from the dead wolf found close to the site.

### Site C

The camera trap recorded one wolf that rested in the haystack at site C throughout the whole recording period (14th15th January from 4 pm to 10 am) with two breaks of 64 and 21 min. The wolf additionally interrupted its rest three times to dig in hay, probably to get to drier hay layers due to heavy snowfall that lasted throughout the night. No other wolves were recorded at site C. No subsequent use of haystacks by wolves was noted during the visits carried out over the following two weeks to check for signs of wolf presence (mostly tracks in the snow).

*S. scabiei* mites were detected in freshly-collected hay samples from both wolf beds, but not in the sample collected two weeks later (Table 1; Fig. 2D). No skin mites were found in the control samples. *S. scabiei* mites were identified in skin scrapings and skin biopsies of both dead wolves found close to the site. Based on the tooth wear, both individuals were less than a year old.

**Table 1:** Detection of *Sarcoptes scabiei* mites in hay samples collected from haystacks used by wolves with skin lesions indicative of sarcoptic mange at three sites (A: Pogorzelce, B: Wolf Glade, C: Stare Masiewo) in Białowieża Primeval Forest (NE Poland) in 2022 and 2024. The data includes the time of sample collection (period) and the time (days) since the last confirmed use of haystack by infected wolves until the date of the sample collection (days Post Wolf observation, DPW). Samples were collected either at the bottoms of the cavities used by the wolves (bed) or on the opposite sides of the same haystacks (control).

Ln	Period	Site	Haystack ID	Sample	DPW	S. scabiei
1	February 2022	Site A	Site A-1	bed	6	*+*
2	February 2022	Site A	Site A-2	bed	6	-
3	May 2022	Site B	Site B -1	bed	45	-
4	May 2022	Site B	Site B -1	bed	45	-
5	May 2022	Site B	Site B -2	bed	45	-
6	May 2022	Site B	Site B -2	bed	45	-
7	May 2022	Site B	Site B -1	control	45	-
8	May 2022	Site B	Site B -1	control	45	-
9	May 2022	Site B	Site B -2	control	45	-
10	May 2022	Site B	Site B -2	control	45	-
11	January 2024	Site C	Site C-1	bed	0	*+*
12	January 2024	Site C	Site C-1	bed	14	*-*
13	January 2024	Site C	Site C-2	bed	0	*+*
14	January 2024	Site C	Site C-1	control	0	-
15	January 2024	Site C	Site C-2	control	0	-

## Discussion

Our findings indicate that wolves with apparent mange symptoms used haystacks for shelter, and that *S. scabiei* mites remained present in the hay for several days after the last time an infected individual had been recorded at the site. Therefore, our findings may have important implications for understanding the spread of re-emerging zoonotic diseases such as sarcoptic mange and may be of value for understanding wildlife-human conflicts.

Wolves with mange were observed to rest in haystacks, which appears to be a behavioural adaptation intended to support their thermoregulation. This behaviour seems intuitive and confirms the behavioural plasticity of wolves, which allows them to use anthropogenic resources, including food and linear features, such as roads and trails^50,51^. Similar warmth-seeking behaviour by wolves with mange has been observed in Wisconsin, USA, where three radio-tracked individuals with advanced sarcoptic mange sought shelter and subsequently died in porcupine (*Erethizon dorstaum*) dens in winter^52^. However, to our knowledge, the present report is the first to indicate that wolves can use manmade structures for shelter and resting sites. It is important to note that while hiding in hay probably did not help the long-term survival of the wolves, as at least three individuals died in the vicinity of haystacks during the study period, it may well have prolonged their lives. Unfortunately, without genetic confirmation it is not possible to confirm whether the dead individuals were the same ones that used the hay. Nevertheless, it seems that wolves used the hay as the last resource, when their overall condition reached a point where they were not able to physiologically thermoregulate.

Moreover, camera recordings of apparently healthy individuals visiting the wolves with mange indicate that the haystack sites served as the pack’s rendezvous sites, where infected young wolves were provided with food by other pack members, a commonly-described behaviour in packs affected by mange^53^. Since two of the sites (site A and site C) were located at the outskirts of settlements, their use stands in contrast with typical behaviour of wolves, which tend to avoid areas of high human use^54,55^. This avoidance usually becomes especially pronounced during the most vulnerable activities, as resting and tending to their young^56,57^, and both these behaviours were displayed close to settlements in this study. Therefore, our findings provide a new potential link between the infection with *S. scabiei* and potentially conflictive behaviour in wolves. Regular use of haystacks located in proximity to settlements may lead to frequent wolf-human encounters, increased consumption of anthropogenic food by wolves and even habituation to human presence^37^. However, predation on domestic animals was not reported during the wolf use of haystacks, even in site C where the wolves had access to a herd of horses. Moreover, the presence of wolves close to houses was not perceived as dangerous by local inhabitants, who seemed to be used to seeing and hearing wolves within and around their villages (authors’ pers. observation). Nevertheless, sarcoptic mange seems to partially suppress the tendency of wolves to avoid humans, thus making them prone to potential conflicts. This aligns with behavioural findings in other mange-affected carnivores, like coyotes and foxes^32,33^.

Haystacks are used by wildlife for a variety of purposes and therefore may become hotspots for intra- and inter-species disease transmission. For instance, wild carnivores of small and medium size, such as the eastern spotted skunk (*Spilogale putorius*) and European badger (*Meles meles*) have been reported to utilize haystacks as shelters, dens or feeding sites^58,59^. In fact, Garnett et al.^59^ found that using haystacks and other farm resources by badgers facilitated transmission of tuberculosis caused by *Mycobacterium bovis* to cattle. The presence of mange mites in haystacks found in this study indicates that similar mechanisms may be present in the case of *S. scabiei*. This possibility is confirmed by our camera-trap recordings, as haystacks were visited also by red foxes with mange symptoms. The red fox is particularly susceptible to sarcoptic mange^22^ and infected individuals often visit human settlements in search of anthropogenic food sources^60^, which makes them an important vector of mange transmission between wild and domestic animals. Also, in our study, the routes of transmission of sarcoptic mange can be multidirectional. The presence of wolves carrying sarcoptic mange close to settlements also increases the probability that domestic animals come into contact with infected wolves or pathogens left in haystacks, which could result in the transfer of the skin parasites. Previous research has reported transmission of *S. scabiei* to domestic animals by wildlife with mange approaching farms, e.g. between chamois (*Rupicapra rupicapra*) and domestic goat^61^ and between wild carnivores and livestock^59,62,63^. However, the direction of transmission pathways is usually opposite, i.e. from domestic animals to wildlife; thus in most of cases, it is the domestic species that act as reservoirs of *S. scabiei*, facilitating the global spread of the parasite and thus contributing to the cross-species transmission to wildlife causing devastating population consequences in some species^20^.

Our findings indicate that mites were present in hay at least six days after the last visit of a scabietic wolf, which indicates that haystacks may contain parasites for prolonged periods, as has been noted for dens^64^. Although it was not possible to test the infectivity of the mites recovered from the hay, it seems likely that environmental transmission may occur in haystacks, due to the long survival of *S. scabiei* away from the host, and its host-seeking behaviour^23,65^. This suggests that haystacks may pose a high risk of inter- and intra- species environmental transmission of sarcoptic mange.

Our findings represent the first record of a manmade structure serving as an environmental reservoir for indirect transmission of sarcoptic mange in wildlife. So far, only natural burrows and dens have been reported as transmission sites for sarcoptic mange^25–27^. While some carnivores use dens and burrows throughout the year and share it among species^66–68^, wolves only use dens for pup rearing and do not share them with other potential mange hosts, which limits the probability of indirect sarcoptic mange transmission from wolves to other species. In contrast, due to their thermo-insulating properties, haystacks may be used by individuals of various species and throughout the cold season, which makes them potentially efficient hotspots for intra- and inter-species transmission. The contribution of far-ranging species like wolves to the environmental transmission of sarcoptic mange may significantly accelerate its spatial expansion. The spread of sarcoptic mange may be further facilitated by global change, as mild winters with increasingly short periods of frost and snow are becoming more common in this latitude, thus providing more suitable conditions for the off-host survival of mites^23,69^. Indeed, our findings confirm that sick individuals used haystacks not only during a frosty and snowy winter (January 2024) but also in a snowless and relatively warm period (March 2022).

The numbers of haystacks, their distribution and the extent to which they are used by wild ruminants depend on local wildlife management strategies. In the BPF, hay is often left over winter to provide supplementary food for European bison. Intentional European bison feeding activities have been conducted in BPF since the 1700s; however, an unintentional system was present even earlier, with haystacks being left on mown meadows^70^. Although the density of feeding sites and the frequency of their supplementation vary among years, haystacks are prepared each year before winter and left until spring. Wildlife supplementary feeding is common in Europe and is mainly intended to reduce the impact of herbivores on agriculture, to enhance their body condition and reproductive performance, or to supply endangered species with food or water^71^. However, supplementary feeding may also play a negative role in wildlife health^6,72,73^. In the BPF, supplementary feeding in fixed locations led to a reduction in European bison home ranges in winter and an increased aggregation, which affected their exposure to parasites and favoured the spread and the severity of parasite infections^74,75^. Our findings indicate that the hay provided as supplementary food for European bison may be also a hotspot of sarcoptic mange transmission for wolves. This may also facilitate transmission of mange to European bison, as *S. scabiei* in BPF has been already documented in this species^76,77^. However, this assumption should be taken with caution and needs further studies, as genetic research suggests that while *S. scabiei* is a single mite species, it likely consists of several genetic variants with variable degrees of host specificity^63,78,79^. No genetic studies appear to have compared the *S. scabiei* from European bison and wolves in this area.

In this study, sarcoptic mange was detected using a novel method: a combination of camera trapping with laboratory analyses of hay samples, verified with skin examination of dead individuals. Disease monitoring and diagnostics in wildlife must often rely on material from dead animals or from their faeces. However, animals suffering from mange have visible skin symptoms that allow the use of camera trapping for monitoring, as demonstrated by several recent studies^33,80–82^. However, these visual methods are limited, as mange-like symptoms may have a different aetiology^83^. Nevertheless, our findings confirm the value of non-invasive methods, i.e. camera traps and analyses of environmental samples (hay), for detection of skin diseases and its causative agents, but also for monitoring the behaviour of infected individuals. This is particularly important as certain behaviours may facilitate the spread of mange by creating hotspots for its transmission. The hay that was sampled two or more weeks after the last confirmed use by wolves did not contain *S. scabiei* mites, which indicates that, in future studies, it is advisable to collect hay samples within the period of regular use by scabietic animals.

## Conclusions

Our findings indicate an unforeseen link between wild herbivore supplementary feeding, transmission of skin diseases, and potentially conflictive behaviour in wolves. This link requires infected wolves to be attracted to haystacks close to settlements, and the accumulation of sarcoptic mange mites in these haystacks. Our data also provide the first example of potential human-facilitated environmental transmission of sarcoptic mange, an emerging wildlife panzootic. Extensive research is still required to ensure effective science-based management of wild populations, particularly when dealing with rare and endangered species which need effective conservation management, such as the European bison.

To fully understand the role of haystacks in transmission of *S. scabiei*, further studies are needed to determine the viability and infectivity of the mites deposited in the hay. Moreover, the use of genetic methods in these studies would provide a further insight into the variation between *S. scabiei* mites obtained from different hosts, such as wolves, foxes and European bison, and those collected from hay. The suggested methodological modifications will allow more accurate evaluation of the risk of interspecies transmission among mammals using the same haystacks as shelters or feeding sites, and thus the degree of risk to wild mammals susceptible to mange.

## Data Availability

Data used during the study are available from the first author (Katarzyna Bojarska, katbojarska@gmail.com) on reasonable request.
